# Editorial: Patient safety in medical physics

**DOI:** 10.1120/jacmp.v11i2.3328

**Published:** 2010-04-16

Recent articles in US newspapers^(^ ^1^ ^–^ ^4^ ^)^ have increased the awareness of the public to the potential dangers of incorrect delivery of medical radiation. In response, many of the leading organizations representing users of medical radiation have joined to identify and address the common issues that could substantially improve the safety and quality of the use of medical radiation. I have invited Dr Michael Herman, President of the American Association of Physicists in Medicine, to share with the JACMP readership some of the initiatives being placed forward to improve medical radiation safety. I trust our readership will find Dr Herman's comments relevant and informative.

George Starkschall, PhD

Editor‐in‐Chief

May 15, 2010
